# Occupational therapy interventions for adult informal carers and implications for intervention design, delivery and evaluation: A systematic review

**DOI:** 10.1177/03080226221079240

**Published:** 2022-04-24

**Authors:** Kerry Micklewright, Morag Farquhar

**Affiliations:** 1 School of Health Sciences, 6106 University of East Anglia, Norwich, UK

**Keywords:** Occupational therapy, caregiver, systematic review, adult, intervention, support

## Abstract

**Introduction:**

Informal carers provide vital support for patients, reducing strain on health and social care services. However, caring can detrimentally affect carers’ health and wellbeing, thus policy advocates for improved carer support. Objective: to establish the published international evidence base regarding interventions for carers delivered by occupational therapists.

**Method:**

English language studies published January 2010–January 2021 were identified against predetermined inclusion/exclusion criteria via searches of MEDLINE, EMBASE, CINAHL, PsychINFO, OTSeeker, Scopus, Web of Science and the Cochrane Library. Supplemental strategies: database alerts, hand-searching, searching of included papers’ reference lists and citations, and contacting key authors. Two reviewers completed critical appraisal and produced a textual narrative synthesis of data using a convergent integrated method.

**Results:**

38 papers were included, reporting 21 interventions. Most were dyadic, home-based interventions for carers of people living with dementia. Common intervention components included: assessment and goal-setting, skill training, education, coping strategies, equipment provision, environmental adaptation and signposting. Interventions improved outcomes for carers, however, intervention design and evaluation require careful consideration to maximise carer benefits and capture intervention effects.

**Conclusion:**

Occupational therapist delivered carer interventions enhance support and improve carer outcomes. Intervention and evaluation designs should include careful selection of outcome measures, avoidance of increased carer burden in dyadic interventions and acknowledgement of known barriers and facilitators to both carer and therapist intervention engagement.

## Introduction

Informal carers are unpaid ‘lay people in a close supportive role who share in the illness experience of the patient and who undertake vital care work and emotion management’ (Thomas et al., 2004). The estimated value of informal care significantly outweighed that of formal care provided via health and social services in 2015/6 and was valued at between £57 and 100 billion a year (Buckner & Yeandle, 2015; Office for National Statistics, 2018). This figure is likely to have increased. Prior to the Covid-19 pandemic, approximately 10% of the UK population – or 6.5 million people (Carers UK, 2019) – were carers. Restrictions introduced to protect those vulnerable to Covid-19, combined with reduced access to formal support, led to an estimated increase of 4.5 million additional informal carers in the UK (Carers Week, 2020). Informal carers play a vital role in facilitating hospital discharges, admission avoidance and enabling patients to remain living at home, thus reducing strain on services. They assist patients in maintaining their independence and wellbeing, provide emotional support and often take on a range of responsibilities including household tasks, meal preparation, managing medical appointments and medication, shopping, financial management and personal care (Carers UK, 2019).

However, providing this care can have detrimental impacts on carers’ health and wellbeing (Foley et al., 2021). During the Covid-19 pandemic, carers reported worsening mental (64%) and physical (58%) health (Carers UK, 2020). Alongside financial difficulties, fatigue, stress and problems balancing caring responsibilities with employment, informal carers also experience reduced happiness, poorer health and increased loneliness when compared to the general population (Carers UK, 2019; Foley et al., 2021; NHS 2019). Despite increasing emphasis on carer identification and support in recent policy, carers continue to report poor health and difficulties accessing support (Department of Health and Social Care, 2018; NHS 2019). Meeting the needs of carers is essential to ensure that they can manage their own health and wellbeing whilst also enabling them to provide care throughout the patient’s illness trajectory, and beyond into bereavement.

Occupational therapists are obligated to contribute to carer support and are well-placed to do so, often working closely with patients’ friends and family (RCOT, 2017). However, a lack of published evidence demonstrating the impact of occupational therapy for informal carers has previously been noted (Hall & Skelton, 2012), which may limit opportunities to develop new interventions and the presence of the profession in relation to carer support. Previous reviews have been completed regarding occupational therapy interventions for informal carers, but have either (a) related to support for particular subsets of carers (for example, specific patient diagnosis such as dementia [e.g. Raj et al., 2021]), (b) described potential interventions delivered by other professionals that could be utilised by occupational therapists (e.g. Hall & Skelton, 2012) or (c) focused on particular outcomes (e.g. Abrahams et al., 2018). Whilst these reviews provide valuable insights into the role and efficacy of occupational therapy interventions for informal carers, a comprehensive synthesis of published literature has not yet been completed.

This review’s objective is to establish the published international evidence base from the previous decade regarding interventions for informal carers delivered by occupational therapists. Adult carers are the focus as they comprise the majority of the UK informal carer population (Foley et al., 2021). The needs of young carers, or parents supporting children, are likely to differ significantly, as are the interventions designed to meet these needs; as such, these groups would benefit from separate reviews. This review considers the nature and breadth of relevant interventions, reported intervention outcomes, quality of eligible studies, barriers and facilitators to carer engagement and experiences of occupational therapists in delivering carer-focused interventions.

## Methods

A systematic search of peer-reviewed English language research literature was completed by two reviewers. The protocol was registered (Prospero database: CRD42020203026, accessible at https://www.crd.york.ac.uk/prospero/display_record.php?RecordID=203026) and the PRISMA checklist used to ensure transparency in reporting (Moher et al., 2009).

### Study identification

Initial scoping searches returned interventions addressing carers’ physical and/or mental health; some related to occupational therapists working independently but some were also working as part of a multi-disciplinary team (MDT). Thus, papers that included distinct intervention by an occupational therapist within a wider group of professionals were considered as long as the contribution of the therapist was clearly defined and directly related to carers. As such, databases spanning multiple disciplines were searched: MEDLINE, EMBASE, CINAHL, PsychINFO, OTSeeker, Scopus, Web of Science and the Cochrane Library. Supplementary strategies included: database alerts, hand-searching (The British Journal of Occupational Therapy, January 2010-January 2021) and searching reference lists and citations of included papers. Hand-searching was utilised to check electronic search terms were comprehensive; results were cross-referenced with papers retrieved via database searches. Key authors (five contacted; two responses) were approached via email to enquire if further publications relating to eligible interventions were imminent and identify additional papers potentially eligible for inclusion. Due to time limitations grey literature was not included, however, scoping searches of Open Grey, ProQuest and Ethos returned minimal material.

Eligible papers were identified against predetermined inclusion/exclusion criteria (detailed in full as supplemental material - Appendix 1). Briefly, eligible papers were: related to adult (18+) informal carers for adult patients; English language; empirical research (qualitative, quantitative or mixed methods); published between January 2010-January 2021; focused on interventions involving direct carer support from an occupational therapist (dyadic papers where occupational therapists directly supported patients only were excluded); and reported on intervention efficacy for carers via identification (qualitative) or measurement (quantitative) of outcome measures. Lower quality evidence such as editorials, opinion pieces, case studies and non-empirical material were excluded to increase strength of findings. Where a full randomised controlled trial (RCT) was available, associated feasibility or pilot studies were excluded so that the strongest level of evidence was included. Where RCTs were not available, feasibility and pilot studies were included to enhance comprehensiveness of the review whilst acknowledging their limitations.

A variety of search terms (see supplemental material Appendix 1) were used for the term ‘carer’ as a previous systematic search by the reviewers identified a broad range of terms in use in papers (Carers UK, 2016; Micklewright & Farquhar, 2020).

After removal of duplicates, the first reviewer (KM) screened titles and abstracts against the inclusion criteria; the full text was read if eligibility was uncertain. If eligibility remained unclear, the paper was discussed with the second reviewer (MF) to reach consensus. Reproducibility of screening was established via review of a random sample (10%) of potentially eligible papers by the second reviewer (MF).

### Data extraction

Data was extracted using a bespoke digital data extraction form, modified following successful utilisation in a previous systematic review (Micklewright & Farquhar, 2020). The reviewers individually extracted data from six (16%) eligible papers and compared findings to ensure a consistent and comprehensive approach. The first reviewer (KM) then completed data extraction for the remaining papers.

### Data synthesis

Given the variety of study designs retrieved, a textual narrative synthesis of extracted data was completed using a convergent integrated method (Joanna Briggs Institute, 2019). Data were subsequently analysed for consistency and divergence between findings, gaps in the literature and trends in methodological strengths and weaknesses across studies.

### Critical appraisal

Critical appraisal was undertaken to assess studies for risk of bias and identify other methodological weaknesses potentially influencing the validity of findings. Appraisal of all included papers was completed by the first reviewer (KM) with a random sample (10%) appraised by the second (MF) to enhance reliability. Appraisal tools used depended on the study design and included:

1) The Critical Appraisal Skills Programme (CASP) suite: specifically, the Randomised Controlled Trial, Qualitative and Economic Evaluation checklists (CASP, 2021).
2) The Mixed Methods Appraisal Tool (MMAT) where a suitable CASP tool could not be found (Hong et al., 2018). The MMAT is a tool specifically developed for use in systematic reviews to facilitate appraisal of quantitative, qualitative and mixed methods studies within the same review.

To maintain comprehensiveness of the review, appraisal was not used to exclude studies; instead, methodological issues identified were analysed to determine the credibility of papers as evidence of an intervention’s efficacy. A standardised strength of evidence framework was not utilised, however, credibility of the body of evidence was considered regarding (a) included study designs, (b) quality of individual studies and (c) consistency of the evidence.

## Results

**Figure 1.**
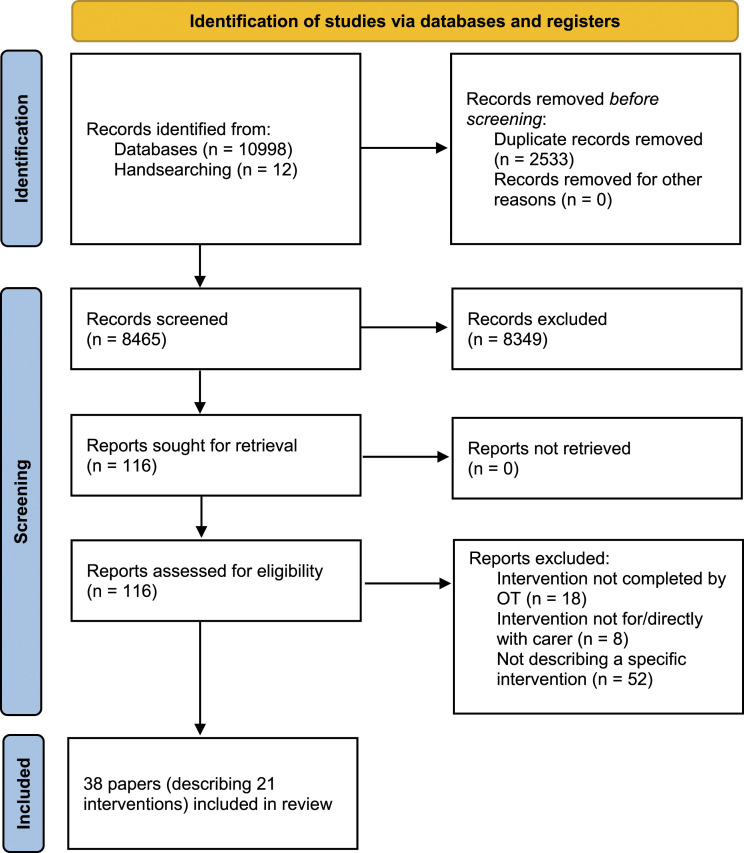
PRISMA flow diagram (based on Page et al., 2021).

Searches yielded 38 eligible papers relating to 21 interventions (see Figure 1 – PRISMA flowchart), completed in: Australia (11), United States (8), Netherlands (4), United Kingdom (4), Brazil (2), Germany (2), Spain (2), Belgium (1), France (1), Italy (1), Hong Kong (1) and Japan (1). Included study designs: RCT (13), feasibility (6), pilot (6), qualitative (4), cost-effectiveness (2), mixed methods (2), process evaluation (2), hybrid implementation-effectiveness study (1), non-inferiority RCT (1) and a retrospective pre-test post-test design (1). An overview of study and intervention designs is available as online supplemental material (see Supplementary Table S1).

### Quality appraisal

For eligible RCTs, the nature of the interventions meant introduction of bias was unavoidable; only individuals collecting outcome data, and independent of intervention delivery, could be blinded to group allocation. Additional potential sources of bias in RCTs included: use of proxies to complete outcome measures, for example, carers (for patient-related outcome measures) or interventionists; attrition (although intention to treat analyses were often used); baseline differences between allocated groups despite randomisation; and intervention fidelity issues. Qualitative studies were largely well-reported, though reflexivity was not always discussed. All study designs appeared appropriate to meet stated aims. Quality appraisal summary tables are available as online supplemental material (see supplemental material Appendix 2).

### Participants

By far the majority of papers described interventions targeting carers of people living with dementia or non-specific cognitive impairment (31). Other patient groups included patients with hip fractures (2), Parkinson’s disease (2), eating disorders (1), cancer (1) and stroke (1). Carer participants tended to be patients’ spouses or adult children; the majority, in most studies, were women. Where reported, average carer age was 55+ years.

### Intervention design

Most interventions were dyadic, addressing both the carer and patient (32 papers/15 interventions); others were carer-only (six papers/five interventions). Several papers reported the use of a pre-established intervention in a new context (e.g. patient group or country): for example, the Community Occupational Therapy in Dementia intervention (COTiD; *n*=6 papers), the Care of Persons with Dementia in their Environments intervention (COPE; *n*=6 papers) and the Tailored Activity Program (TAP; *n*=4 papers).

Interventions offered support directly for the carer or support to facilitate caring, a distinction previously discussed elsewhere (Stajduhar et al., 2008); some addressed both aspects. Common components included: assessment of carer needs/concerns (using a variety of methods) and goal-setting; education and skill training (including: condition-specific knowledge [e.g. symptom management], positive risk-taking, task supervision, compensatory strategies, medication management, environmental adaptation, communication techniques, facilitation of specific activities of daily living [ADLs], helpful aids/equipment, problem-solving and task simplification); hands-on demonstration (e.g. manual handling); coping/stress management techniques; provision of adaptations, assistive technology and aids to facilitate caring; and signposting. Intervention periods spanned from one-off sessions to two years. The majority of papers described home-based interventions using home visits (28); others included group workshops on clinical sites (6), outpatient clinics (2), case-management (1) and ward-based information provision with telephone follow-up (1).

Most papers described occupational therapists as the sole interventionists (21). Others stated additional healthcare professionals involved in intervention development and delivery including nurses (*n*=7 papers), physiotherapists (4), therapy assistant practitioners (2), psychologists (2), social workers/welfare practitioners (2) geriatricians (1), neurologists (1), neuropsychologists (1), nursing assistants (1) and orthopaedic surgeons (1).

### Outcome measures

Various outcome measures were used to evaluate intervention efficacy (see Table 1). In quantitative or mixed-method papers, measured concepts included: patients’ condition-specific symptoms or behaviours (e.g. the Parkinson’s Disease Questionnaire-39); carer/patient quality of life; carer depression, anxiety, pain and fatigue; ease of ADL completion; carer general health; and concepts focused on the carer role such as burden, mastery, upset, stress, strain and confidence. In dyadic interventions, patient outcomes predominated, with some papers including carer-specific measures as secondary outcomes only. Among carer-specific outcomes, the Zarit Burden Interview was the most commonly used, reported in 11 papers. Some study investigators developed their own measures, such as questionnaires to gauge patient/carer satisfaction. Several papers gathered qualitative data, either as the primary method of evaluation or to enhance understanding via interviewing a sub-sample of participants from large-scale RCTs. Semi-structured interview was the primary method of qualitative data collection; inductive thematic analysis the most commonly used analytic approach.

**Table 1.** Outcome measures used in eligible studies. BID: Beck Depression Inventory; BADLS: Bristol Activities of Daily Living Scale; CES-D: Center for Epidemiologic Study Depression Scale; CMI: Caregiving Mastery Index; COPM: Canadian Occupational Performance Measure; CSI: Caregiver Strain Index; EQ5D: EuroQol five dimensions; EDSIS: Eating Disorders Symptom Impact Scale; GAD-7: Generalized Anxiety Disorder 7-item; GAS: Goal Attainment Scaling; GHQ-28: Goldberg General Health Questionnaire; GADS: Goldberg Anxiety and Depression Scale; HADS: Hospital Anxiety and Depression Scale; NPI: Neuropsychiatric Inventory; PCI: Perceived Change Index; PWI-A: Personal Well-Being Index for Adults; QOL-AD: Quality of Life Scale in Alzheimer’s disease; RUD: Resource Utilisation in Dementia; RSS: Relatives Stress Scale; SCQ: Sense of Competence Questionnaire; SF-12: Short-Form 12 Health Survey Questionnaire; TMSI: Task Management Strategy Index; WHOQOL-BREF: The World Health Organisation Quality of Life-Brief; UPCC: Utrecht Proactive Coping Competence Scale; ZBI: Zarit Burden Interview. Note: papers reporting process evaluations or additional outcomes from the same study are presented together with the original paper.

To right: Papers Below: Outcome measures	Gitlin et al. (2010a)	Gitlin et al. (2018)	de Oliveira et al. (2018)	Novelli et al. (2018)	O’Connor et al. (2019)	Van’t Leven et al. (2011)	Voigt-radloff et al. (2011a/2011b)	Pozzi et al. (2019)	Wenborn et al. (2021)	Burgess et al. (2020)	Donkers et al. (2018)	Gitlin et al. (2010b)	Clemson et al. (2020)	Culph et al. (2020)	Rahja et al. (2020a)	Rahja et al. (2020b)	Fortinsky et al. (2020)	Laver et al. (2020)	Lam et al. (2010)	Eames et al. (2013)	Pépin & King (2013)	Wesson et al. (2013)	Martín-Martín et al., (2014)	Sturkenboom et al. (2014/6)	Callahan et al. (2017)	DiZazzo-Miller et al. (2017/20)	Nishida et al. (2017)	Cornelis et al. (2018)	Corvol et al. (2018)	Allan et al. (2019)	Clare et al. (2019)	Ariza-Vega et al. (2020)	Jeon et al., (2020)	Morency et al. (2020)	O’Connor et al. (2019)
	TAP					COTiD (and SPF)						COPE							Other interventions																
ADL knowledge test																										X									
BADLS																										X									
BDI																										X									
Caregiver confidence scale																										X									
Caregiver vigilance scale (vigilance items)					X																														
CES-D		X																																	
CMI																		X																	
COPM																								X		X									
Cost	X															X																			
CSI																				X															
EQ5D (and variants)																								X								X	X		
GAD-7																									X										
GADS																							X												
GAS																														X					
GHQ-28																							X												
HADS									X															X											
Investigator-developed measure(s)												X	X		X		X	X		X	X											X		X	
Knowledge of stroke questionnaire																				X															
NPI				X																															
NPI-C (Clinician-completed)		X															X																		
PCI												X	X				X	X																	
PWI-A																			X																
QOL-AD								X																											
Qualitative data					X					X	X				X														X		X		X	X	X
Quality of life scale				X																															
RUD							X																												
RSS																															X				
SCQ							X	X	X																										
SF-12							X	X																											
TMSI																						X													
The brief COPEEDSIS																					X														
WHOQOL-BREF																										X					X				
UPCC																								X											
ZBI		X	X	X				X											X			X		X			X	X		X			X		

### Intervention effects

#### Quantitative carer outcomes

*Home-based interventions:* Papers related to the TAP intervention consistently reported positive carer outcomes. The original study, which was not formally part of this review due to publication more than a decade prior, reported significant increases to self-perceived mastery and self-efficacy (Gitlin et al., 2008). A later follow-up RCT in the same country (USA) described significant reduction in carer distress with patients’ behavioural symptoms and an improvement trend for other outcomes (Gitlin et al., 2018). Pilot and feasibility studies in Brazil and Australia reported positive outcomes (decreased carer burden and increased quality of life) and good carer engagement, respectively, although with limited sample sizes these must be considered with caution (Novelli et al., 2018; De Oliveira et al., 2018).

COTiD, in contrast, has shown limited evidence of efficacy beyond the Netherlands where effectiveness was first demonstrated in terms of multiple carer-related outcomes and cost (Graff et al., 2007). No significant differences in quantitative outcome measures were reported in subsequent German and UK trials (although positive qualitative data was reported from the latter) (Voigt Radloff et al., 2011a, 2011b; Wenborn et al., 2021). Another attempt at adapting COTiD with additional physiotherapy and social work components resulted in a failed trial (the Social Fitness Programme); a parallel process evaluation suggested numerous barriers to intervention delivery and significant recruitment difficulties (see Supplementary Table S1) (Donkers et al., 2018). An Italian feasibility study reported a significant difference in carers’ sense of competence post-COTiD, though with a small sample size (*n* = 27) it is unclear whether a similar result would be achieved in an RCT (Pozzi et al., 2019).

The original COPE intervention trial reported significant increases in carer wellbeing and confidence (Gitlin et al., 2010b); subsequent trials in Australia and USA reported significant improvement in carer-perceived changes (on the Perceived Change Index) (Clemson et al., 2020; Fortinsky et al., 2020). A ‘non-inferiority RCT’ reported an adapted COPE intervention using telehealth input from occupational therapists was not inferior to face-to-face delivery (Laver et al., 2020).

Other significant carer-related outcomes reported from home-based interventions included: improved carer quality of life at three months (Occupational Therapy in Parkinson’s Disease/OTiP, RCT [Sturkenboom et al., 2014, 2016]) and significant increase in carer health-related quality of life (the Home-based Reablement Program/I-HARP, pilot study [Jeon et al., 2020]). The Alzheimer’s Disease Multiple Intervention Trial by Callahan et al. (2017) (ADMIT, RCT), a pilot study by Nishida et al. (2017) and the Developing an Intervention for Fall-Related Injuries in Dementia (DIFRID, feasibility study) by Allan et al., (2019) reported no significant differences between groups for positive carer outcomes.

Notably, multiple papers describing dyadic, home-based interventions reported significantly increased carer burden in comparison to control groups, including DIFRID, I-HARP and a feasibility study of a falls-prevention programme (Wesson et al., 2013) – the latter reporting an almost doubling of carer burden for the intervention group. The majority of studies also reported that even when a positive outcome was achieved, it was often not maintained long-term; researchers speculated that this may be due to changes in the health and functioning of patients (and by extension, the carers’ needs) over time.

*Other interventions:* An RCT of a case-management intervention (occupational therapist assessment and treatment of dyads from various outpatient clinics), found no significant changes in outcomes, although use of social care support increased in the intervention group; the author considered this a positive sign that dyads were more willing to accept help and had been successfully signposted to these resources (Lam et al., 2010). A multicomponent rehabilitation programme based in an outpatient clinic reported non-significant outcome changes for carers but stated that 60% showed improved or stable burden and distress by programme end (Cornelis et al., 2018).

More positive findings were associated with an RCT of a pre-discharge educational training programme for the carers of new hip fracture patients, with greater decreases in anxiety and depression over time for intervention group carers (Martín-Martín et al., 2014). A feasibility study of another hip fracture instructional workshop was also well-received, with evidence of improved carer knowledge in relation to delivering care following a hip fracture (Ariza-Vega et al., 2020). An educational programme for dementia carers reported improved knowledge (in relation to a range of topics) and physical health in intervention carers, although sample sizes were small (DiZazzio-Miller et al., 2017; 2020). A pilot study examining the transferability of the UK-developed Collaborative Care Skills Training workshop to Australia reported significant increases in use of adaptive coping strategies post-programme and trends towards improved carer outcomes for carers of people living with eating disorders (*n* = 15) (Pépin & King, 2013). An RCT of a ward-based educational intervention for stroke patients and their carers reported no significant carer outcomes (Eames et al., 2013). Supplementary Table S1 (supplementary material) provides further information on carer-related intervention outcomes.

### Qualitative carer outcomes

Qualitative feedback from carers was almost always positive. Carers described how interventions facilitated positive interactions with the patient (Corvol et al., 2018; O’Connor et al., 2019), a sense of being in control (O’Connor et al., 2019), new skills and knowledge (and that these were useful) (Burgess et al., 2020; Clare et al., 2019; Rahja et al., 2020a), increased patient confidence and independence (which they appreciated) (Corvol et al., 2018) and reported their enjoyment of therapeutic relationships with occupational therapists (Burgess et al., 2020; Clare et al., 2019). Carers appreciated flexibility in the timings of intervention components (e.g. home visits or individual sessions) (Morency et al., 2020), consideration of, and tailoring to, their particular situation (Morency et al., 2020), continuity (Jeon et al., 2020), advice being specialist but accessible (Jeon et al., 2020), good communication from interventionists (Burgess et al., 2020) and validation from professionals regarding their caring role (Rahja et al., 2020a).

However, while carer engagement was generally high, some studies identified barriers, reported by interventionists, study investigators or carers themselves. These included: time pressures (for carers) (O’Connor et al., 2019; Voigt-Radloff et al., 2011b), high carer stress/worry (Jeon et al., 2020), difficulties within patient-carer relationships (Clare et al., 2019; Voigt-Radloff et al., 2011b), carers feeling unable to talk about patients in front of them (Jeon et al., 2020), low carer belief in potential intervention effectiveness (Voigt-Radloff et al., 2011b), unwillingness to accept change or support (Donkers et al., 2018; Voigt-Radloff et al., 2011b), and reluctance to address actual or potential patient disease progression (O’Connor et al., 2019). Sometimes professionals acted as barriers by gatekeeping: one paper described how professionals, wary of increasing carer burden, felt reluctant to refer carers to the intervention study despite its potential benefits (Donkers et al., 2018). Another noted that although carer support had been an intended intervention component, it was often neglected by interventionists due to a lack of operational detail in the protocol (Allan et al., 2019). Other papers reporting on dyadic interventions noted that, on occasion, carers would use intervention sessions as respite despite the intention that activities would be completed jointly with the patient, perhaps emphasising the time pressures and lack of day-to-day respite carers can access (Corvol et al., 2018).

No known harmful effects were reported as a result of intervention participation in any of the papers.

### Occupational therapists’ experiences

Of the papers that reported feedback from interventionist occupational therapists, therapists appeared to value the opportunity to participate (Burgess et al., 2020). They appreciated being given time to practice core skills (‘real OT’) and form deeper relationships with patients and carers (Burgess et al., 2020). Facilitators to occupational therapist participation included: confidence in their skills (Van’t Leven et al., 2011); intervention familiarity (Van’t Leven et al., 2011); managerial support (Culph et al., 2020; Van’t Leven et al., 2011); belief the intervention was useful for patients and carers (Van’t Leven et al., 2011); and positive relationships with other MDT members (where applicable), bolstered by shared working environments (Culph et al., 2020). Barriers included: intervention elements or procedures perceived as overly-complex (Van’t Leven et al., 2011); time pressures (Van’t Leven et al., 2011); low patient/carer motivation (Donkers et al., 2018; Voigt-Radloff et al., 2011a); delays in accessing onward services, community resources or equipment (Burgess et al., 2020); and poor communication between/access to other MDT members (Culph et al., 2020; Donkers et al., 2018). Therapists commented on the importance of carer involvement in dyadic interventions and impact of dyads’ relationships on intervention success (Burgess et al., 2020; Clare et al., 2019).

### Cost

Reporting of financial costs was variable but suggested potential financial benefits from adopting evaluated interventions on a larger scale. Gitlin et al. (2010a) found the TAP intervention costed less than similar contemporary dyadic interventions, with reduced time per day spent caring; Rahja et al. (2020a) similarly found COPE implementation reduced carer time away from employment, potentially benefitting the Australian health and social care system. Clare et al. (2019) suggested functional gains via the GREAT intervention could save health and social care costs via patients’ functional gains if Willingness-To-Pay values were ≥£2500.

## Discussion

This review aimed to establish the international published evidence base relating to interventions for adult informal carers delivered by occupational therapists. Findings indicate a range of interventions have been developed and evaluated, utilising a variety of intervention designs to improve carer outcomes. While outcomes varied between individual papers, the evidence suggests the overall impact of occupational therapy for carers through these interventions is positive, that carers value the input of occupational therapists and that occupational therapy could be a safe and cost-effective option for improving carer support. Additionally, therapists themselves appear to enjoy delivering these interventions and the opportunity to use core professional skills.

One major finding of the review is the importance of carefully considering outcome measures to ensure intervention effects are successfully captured. Another key finding was that success of an intervention in one context does not guarantee the same when introduced elsewhere. Outcomes indicated the efficacy of interventions varied by country: for example, despite significant success in the Netherlands where it originated (Graff et al., 2007), COTiD outcomes suggested reduced efficacy in subsequent German and UK RCTs (Voigt-Radloff et al., 2011a; Wenborn et al., 2021). The reasons for this appear complex. A process evaluation for the German RCT linked poorer outcomes to various possible explanations, including better baseline functioning of patients than the original study (Voigt-Radloff et al., 2011b). The COTiD UK team received very positive qualitative feedback from participating dyads, but quantitative outcome measures indicated COTiD was no more effective than treatment as usual; the authors questioned if, rather than the intervention being ineffective, the selected outcome measures (which differed from the original COTiD RCT) were not appropriate to detect intervention effects (Wenborn et al., 2021). In the case of two large trials (Clare et al., 2019; Wenborn et al., 2021), qualitative data proved useful in detecting intervention effects where quantitative outcome measures did not, suggesting qualitative components should be included in evaluations.

Interestingly, the outcome measure most frequently used was the Zarit Burden Interview (ZBI) (Zarit et al., 1980). The ZBI can be a useful outcome measure but its use and interpretation should be considered carefully, taking into account the potential for patient deterioration; most papers related to interventions for carers of people with dementia, wherein symptom severity (and hence carer burden) is likely to increase over time regardless of health or social care intervention. While most interventions lasted for a period of months, evaluation often continued for up to a year after intervention commencement, a timeframe in which the needs of patients could conceivably change and intensify. Cornelis et al. (2018) considered stable ZBI scores to indicate a positive effect on dementia carers given the extended length of their intervention (maximum 12 months).

In anticipation of deterioration, some interventions included components related to preparing carers for the future (e.g. the TAP and COPE interventions) however effects were not always maintained longer term. Maintenance of positive effects post-intervention may be an important factor for intervention developers to consider, though achievement of a positive outcome in the short-term (and hence meeting the immediate needs of a carer) without demonstrating long-term maintenance still contributes to improved carer support and is not without value. Evidence that effects are not always maintained post-intervention may reflect that the needs of carers can change over time in response to a range of factors (e.g. lifestyle change or disease progression).

The majority of papers described dyadic, home-based interventions. This review suggests that care must be taken when designing dyadic interventions to avoid unduly increasing burden on carers. Professionals in a multi-disciplinary feasibility study by Allan et al. (2019) suggested joint initial assessment by professionals to reduce duplication and avoidance of complex or overly long outcome measures, which carers sometimes must also complete on behalf of the patient where insight is unreliable (Allan et al., 2019). Interestingly, poor implementation of carer support was also noted in this paper, despite being an intended component; researchers attributed this to a lack of clarity about how professionals should deliver this. This implies that (a) therapists must have clear understanding of how to operationalise carer support for it to be effective, (b) care must be taken to ensure interventions intended to support a dyad do not inadvertently transition into becoming primarily patient-focused and (c) that while use of the ZBI should be well-considered in terms of outcomes, it can also help as a process measure, detecting when an intervention design is burdensome for carers. Benefits of a dyadic approach can include patients and carers working together constructively, deepened understanding, acknowledgement of each other’s needs and improved relationships (Clare et al., 2019; Corvol et al., 2018; O’Connor et al., 2019); however, carers can also struggle to express themselves in front of patients (Jeon et al., 2020), pre-existing relationship dynamics can affect intervention success (Voigt-Radloff et al., 2011b; Clare et al., 2019) and carers can disengage, misunderstanding the purpose of therapeutic sessions, instead treating them as respite (Corvol et al., 2018). This last point is particularly pertinent given the reported importance of carer engagement for the success of dyadic interventions (Burgess et al., 2020; Clare et al., 2019).

One paper described a failed trial of a dyadic intervention (Donkers et al., 2018) while another presented a trial of a previously successful Dutch intervention which did not yield any improved outcomes in a different context (Voigt-Radloff et al., 2011a). In both cases, process evaluations proved valuable in analysing why these interventions were not successful (see Supplementary Table S1). Similarly, papers describing implementation and staff perspectives on barriers and facilitators to intervention delivery provided useful insights to inform future intervention development and study design. When designing evaluations, capture and publication of this valuable data should be considered to provide transparency and help guide future research.

### Limitations

Though measures were taken to enhance the methodological quality of this review, limitations remain. Exclusion of non-English language papers may mean relevant papers were missed. Additionally, though minimal relevant material was found during scoping searches of grey literature, its exclusion may have increased publication bias (Paez, 2017). The decision to limit searches to the last decade was made after scoping searches; this pragmatic choice ensured completion of the review within the project timescale given the volume of papers retrieved. This meant papers reporting the original RCTs of some interventions fell outside of the given timeframe and hence were not included. However, these papers were read and considered during data synthesis to ensure reviewers held the necessary understanding of intervention development and any previous outcomes associated with them.

The review eligibility criteria meant some papers were excluded that may have contributed useful knowledge but did not fulfil all requirements for inclusion; for example, some papers indicated occupational therapists were involved in an intervention as part of an MDT, but their role or specific contributions were unclear. Use of the TIDieR checklist to enhance clarity in intervention reporting may help ensure future papers are not excluded on a similar basis (Hoffman et al., 2014).

Some of the papers in the review were pilot or feasibility studies. These were included to ensure comprehensiveness of interventions reported in the literature. However, these studies are rarely sufficiently powered to enable definitive conclusions about the effects of an intervention (nor are they designed to do so), or whether they will deliver similar outcomes in a subsequent RCT; as such their efficacy findings should be treated with caution (Thabane et al., 2010).

Finally, papers relating to interventions for young carers were excluded. The needs of young carers are likely to differ from adult carers and would benefit from a separate review. Similarly, many papers were discovered during scoping searches relating to supportive occupational therapy interventions for parents or other individuals caring for children but fell outside the remit of this review. Subsequent reviews synthesising these studies may provide further valuable insight into the impact of occupational therapy interventions for informal carers.

## Conclusion

Informal carers play a vital role in supporting patients, but improved carer support is required to ensure carers can manage their own health and wellbeing alongside providing support for patients. Occupational therapist delivered interventions can play a key role enhancing support for informal carers and improving outcomes, although intervention and evaluation design should be carefully considered, drawing upon lessons learned from the existing international evidence base. This review provides evidence that occupational therapy interventions are a safe and potentially effective option for improving carer support.

**Key findings**

• Occupational therapy interventions can successfully improve outcomes for informal carers

• Outcome measures must be considered carefully to ensure intervention effects are successfully captured

• When designing dyadic interventions, care must be taken to ensure burden on carers is not increased

• A variety of barriers and facilitators affect carer engagement and intervention delivery by occupational therapists

**What this paper adds**

This review synthesises international data from the past decade relating to carer-targeted interventions delivered by occupational therapists. Carer support is increasingly emphasized as a priority for health and social care services; this review provides evidence that occupational therapy is a potentially effective option for improving carer support but suggests that intervention design and evaluation must be carefully considered to achieve this.

## Supplemental Material

sj-pdf-1-bjo-10.1177_03080226221079240 – Supplemental Material for Occupational therapy interventions for adult informal carers and implications for intervention design, delivery and evaluation: A systematic review Supplemental Material, sj-pdf-1-bjo-10.1177_03080226221079240 for Occupational therapy interventions for informal carers and implications for carer support: A systematic review occupational therapy interventions for informal carers by Kerry Micklewright and Morag Farquhar in British Journal of Occupational Therapy

sj-pdf-2-bjo-10.1177_03080226221079240 – Supplemental Material for Occupational therapy interventions for adult informal carers and implications for intervention design, delivery and evaluation: A systematic review Supplemental Material, sj-pdf-2-bjo-10.1177_03080226221079240 forOccupational therapy interventions for adult informal carers and implications for intervention design, delivery and evaluation: A systematic review by Kerry Micklewright and Morag Farquhar in British Journal of Occupational Therapy

sj-pdf-3-bjo-10.1177_03080226221079240 – Supplemental Material for Occupational therapy interventions for adult informal carers and implications for intervention design, delivery and evaluation: A systematic review Supplemental Material, sj-pdf-3-bjo-10.1177_03080226221079240 for Occupational therapy interventions for adult informal carers and implications for intervention design, delivery and evaluation: A systematic review by Kerry Micklewright and Morag Farquhar in British Journal of Occupational Therapy
