# Supplementary material for: Occupational therapy interventions for adult informal carers and implications for intervention design, delivery and evaluation: A systematic review
Source: Br J Occup Ther. 2022 Apr 24;86(2):90–100. doi: 10.1177/03080226221079240 (PMC12033786; doi:10.1177/03080226221079240)
Supplement: sj-pdf-1-bjo-10.1177_03080226221079240 – Supplemental Material for Occupational therapy interventions for adult informal carers and implications for intervention design, delivery and evaluation: A systematic review [file sj-pdf-1-bjo-10.1177_03080226221079240.pdf]

Inclusion criteria

Inclusion criteria	Rationale
Papers relating to interventions that target carers specifically (or if for patient-carer dyads, independently evaluate the effect of the intervention on the carer)	The review is intended to focus on interventions for carers and their effect on the carer themselves
Papers/studies that relate to adult carers supporting adult patients	Interventions primarily targeting a) adult carers for children and b) young carers necessitate separate reviews. Potential areas for intervention and how interventions are conducted are likely to vary considerably between these groups
Papers including interventions where outcomes were measured (quantitative) or identified (qualitative)	This review aims to provide information to show how outcomes were measured or identified and the efficacy of the interventions examined
Interventions that involve occupational therapists	The purpose of the review is to identify interventions led by occupational therapists or including a defined occupational therapy component in order to inform future practice
Editorials, opinion pieces, case studies and non-empirical material	The review will exclude lower quality evidence to increase the strength of the findings
Interventions for informal/unpaid carers	The focus of the review is informal carers. The needs – and potential interventions to address these needs – are likely to differ between informal carers and paid care staff due to differences in their caring roles and their relationship to the patient
English language only	Papers will be in English only due to constraints in time (the PI also holds a part time clinical role as an OT) and to ensure the effective use of resources
Empirical research papers (qualitative, quantitative and mixed method studies)	The review aims to be as comprehensive as possible
Papers from 2010-present	This review aims to cover contemporary research to ensure its relevance to current practitioners/those involved with occupational therapy services. Furthermore, any recent changes to national healthcare policies in relation to how healthcare professionals support informal carers are more likely to be reflected in research from this period.

This review aims to establish:
a) the extent to which occupational therapists are involved in interventions targeting adult informal carers as described within research literature
b) the quality of research evidence that has been produced relating to these interventions
c) the nature and breadth of these interventions
d) information relating to the efficacy of these interventions reported within the literature.

The review aims to focus primarily on interventions led or delivered by occupational therapists. However, it is acknowledged that occupational therapists often work as part of a multi-disciplinary approach. As such, papers that included a distinct intervention by an occupational therapist within a wider group of professionals were also considered as long as the contribution of the therapist was clearly defined and directly involving carers. For example, a study examining a multidisciplinary intervention for carer-patient dyads was excluded if the occupational therapy element only involved the patient. There was no specific threshold for intensity/frequency of therapeutic input by occupational therapists required in multidisciplinary studies for inclusion but occupational therapists had to be delivering a component specifically targeting carers and their role within this intervention had to be considered clear. Studies where carers were included solely to facilitate improved outcomes

for the patient (for example, assisting patients to complete an exercise/rehabilitation programme) without clear incorporation of a well-defined component designed to address carer-specific needs were not considered; the review aims to provide insight into the contribution of occupational therapists to carer-specific support (though it is acknowledged that carers may find participation in such programmes supportive through improvement of patient wellbeing and/or independence).

For further detail, please see the **PICOTS** below:
P (Population): Adult informal carers for adult patients (18+ years of age)
I (Intervention): Occupational therapy interventions developed to improve outcomes for/wellbeing of informal carers
C (Comparator): Standard care (where comparator present)
O (Outcomes): Any outcome measure used to demonstrate the efficacy/effect of the intervention including measures of quality of life, health (physical or psychological, e.g. measures of anxiety, depression, physical disability), carer-specific outcomes (e.g. carer strain or burden), process or outcome data related to the delivery of the intervention or the experience of the occupational therapist delivering it (e.g. staffing implications, views of the therapist in relation to delivering the intervention). Both quantitative and qualitative data will be included
T (Time): Interventions of any length will be considered
S (Study Design): Qualitative or quantitative empirical research

Search terms used in database searching

**Search terms**		
Profession		Population<br>*'OR' between terms*
(IN TITLE OR ABSTRACT)<br><br>Occupational therap*<br>(Allows occupational therapy, occupational therapist, occupational therapists)	**<u>AND</u>**	(IN TITLE OR ABSTRACT)<br>Carer* (allows carers)<br>Caregiver* (allows caregivers)<br>Supporter* (allows supporters)<br>Informal<br>Famil* (allows family, families, familial)<br>Lay* (allows layman, laymen)<br>Spous* (allows spouses, spousal)
Expanders: similar terms *		
Limiters: 2010-2021, English language, adult carers for adult patients only, interventions delivered by occupational therapists		
