# Supplementary material for: Occupational therapy interventions for adult informal carers and implications for intervention design, delivery and evaluation: A systematic review
Source: Br J Occup Ther. 2022 Apr 24;86(2):90–100. doi: 10.1177/03080226221079240 (PMC12033786; doi:10.1177/03080226221079240)
Supplement: sj-pdf-2-bjo-10.1177_03080226221079240 – Supplemental Material for Occupational therapy interventions for adult informal carers and implications for intervention design, delivery and evaluation: A systematic review [file sj-pdf-2-bjo-10.1177_03080226221079240.pdf]

Table A: Summary of quality appraisal using CASP tool for qualitative studies.

Selected articles	Was there a clear statement of the aims of the research?	Is a qualitative methodology appropriate?	Was the research design appropriate to address the aims of the research?	Was the recruitment strategy appropriate to the aims of the research?	Was the data collected in a way that addressed the research issue?	Has the relationship between researcher and participants been adequately considered?	Have ethical issues been taken into consideration?	Was the data analysis sufficiently rigorous?	Is there a clear statement of findings?	Appraisal summary
Van't Leven et al. (2011)	Yes	Yes	Yes	Yes	Yes	Can't tell	Can't tell	Yes	Yes	Data saturation, ethical approval/issues and examination of the researcher's own role not discussed. However, design appropriate for aims, findings clear and paper valuable re: insight into study implementation.
Burgess et al. (2020)	Yes	Yes	Yes	Yes	Yes	Yes	Yes	Yes	Yes	Well-designed and well-reported qualitative paper.
Rahja et al. (2020)	Yes	Yes	Yes	Yes	Yes	Can't tell	Yes	Yes	Yes	Data saturation and examination of the researcher's own role not discussed. However, design appropriate for aims, findings clear and paper valuable re: insight into intervention participation.
Corvol et al. (2018)	Yes	Yes	Yes	Yes	Yes	Can't tell	Yes	Yes	Yes	Examination of the researcher's own role not discussed in detail. May have benefitted from additional reporting re: data analysis.

Table B: Summary of quality appraisal using CASP tool for randomised controlled trials (RCTs).

Selected articles	Did the study address a clearly focused research question?	Was the assignment of participants to interventions randomised?	Were all participants who entered the study accounted for at its conclusion?	Blinding – were participants (P), investigators (I) and assessors (A) blinded?	Were study groups similar at the start of the RCT? (Carers)	Apart from the intervention, were each group treated equally?	Were the effects of the intervention reported comprehensively?	Was the precision of the estimate of the intervention/treatment effect reported?	Do benefits of the intervention outweigh the harms/ costs? (Carers)	Appraisal summary/comments
Randomised controlled trials										
Callahan et al. (2017)	Yes	Yes	Yes	P – No<br>I – No<br>A - Yes	Yes	Yes	Yes (Though difficult initially to separate out carer outcomes)	Yes	No particular benefit or harm found	Cannot discount possible bias as single-blind. Intervention was vs. TAU. Had to use study protocol to clarify carer outcome measures.
Clare et al. (2019)	Yes	Yes	Yes	P – No<br>I – No<br>A - Yes	Yes	Yes	Yes	Yes	Quantitative data demonstrated no benefit; qualitative suggests helpful	Intervention was vs. TAU. Cannot discount possible bias as single-blind. For primary outcomes, patient and carer report was accompanied by therapist report of goal attainment.
DiZazzo-Miller et al. (2017)/(2020)	Yes	Yes	No attrition reported	P – No<br>I – No<br>A - Unclear	Some group differences re: gender ratio and age.	Yes – intervention later repeater for control group	Yes	Yes	No harm found; evidence of gains to QoL and knowledge	Unclear reporting re: who gathered outcome data. If this was the interventionists, they would not have been blinded to group allocation. Small sample sizes, researchers justify by stating sample size decision made using a power analysis calculated using data from their feasibility study. Combined with 2020 paper which reports a further outcome from the original study.
Donkers et al. (2018)	Yes	Yes	N/A - Trial not completed	P – No<br>I – No<br>A - Yes	Yes (until trial ended prematurely)	Yes	Yes	Yes	Trial incomplete. No harms apparent	Trial did not complete – paper largely describes process evaluation. Utilised protocol to supplement this where methodology not fully reported in main

Selected articles	Did the study address a clearly focused research question?	Was the assignment of participants to interventions randomised?	Were all participants who entered the study accounted for at its conclusion?	Blinding – were participants (P), investigators (I) and assessors (A) blinded?	Were study groups similar at the start of the RCT? (Carers)	Apart from the intervention, were each group treated equally?	Were the effects of the intervention reported comprehensively?	Was the precision of the estimate of the intervention/treatment effect reported?	Do benefits of the intervention outweigh the harms/ costs? (Carers)	Appraisal summary/comments
										paper (provided as additional file). Intended to be intervention vs. TAU, then control group to receive intervention after last assessment.
Eames et al. (2013)	Yes	Yes	Yes	P – No<br>I – No<br>A - Yes	Largely, intervention group slightly younger on average	Yes	Yes	Yes	No particular benefit or harm found	Intervention was vs. TAU. Cannot discount possible bias as single-blind. Some positive outcomes for patients, but no particular significant outcomes for carers (burden only carer-specific outcome measure).
Fortinsky et al. (2020)	Yes	Can't tell - (randomised but method unclear)	Yes (modified intent to treat analysis used)	P – No<br>I – No<br>A - Yes	Unclear - more female carers in intervention group; higher levels of depressive symptoms in control group	Mostly – effect of care managers using COPE prescriptions unknown?	Yes	Yes	Small but significant benefits; however any adverse effects (if any) not clearly reported	Intervention compared to treatment as usual. Majority of outcomes relied on patient/carer report (and may have introduced bias). Cannot discount possible bias as single-blind.
Gitlin et al. (2010)	Yes	Yes	Yes	P – Unclear (unlikely?)<br>I – No<br>A - Yes	Unclear	Yes	Yes	Yes	Small benefit but no adverse effects	Cannot discount possible bias as single-blind. Majority of outcomes relied on carer report (and may have introduced bias). Control group intervention used.
Gitlin et al. (2018)	Yes	Can't tell	Yes	P – No<br>I – No<br>A - Yes	Yes	Yes	Yes	Yes	Yes	Cost-effectiveness of COPE has been investigated elsewhere. Cannot discount possible bias as single-blind. Control group intervention used.
Lam et al. (2010)	Yes	Yes	Yes	P – No<br>I – No<br>A - Yes	Smaller control group but	Control group received	Yes	Yes	No harm found; minimal	May have benefitted from more detailed reporting of what the home visit the control group received consisted of.

Selected articles	Did the study address a clearly focused research question?	Was the assignment of participants to interventions randomised?	Were all participants who entered the study accounted for at its conclusion?	Blinding – were participants (P), investigators (I) and assessors (A) blinded?	Were study groups similar at the start of the RCT? (Carers)	Apart from the intervention, were each group treated equally?	Were the effects of the intervention reported comprehensively?	Was the precision of the estimate of the intervention/treatment effect reported?	Do benefits of the intervention outweigh the harms/ costs? (Carers)	Appraisal summary/comments
					demographics appear relatively similar	one visit for home safety, otherwise the same			benefits	Cannot discount possible bias as single-blind. One case manager for 59 dyads for 4 months – median number of follow-ups by type are reported but would be interesting to know how long was spent for each and if intensity of intervention was felt to be feasible with only one case manager.
Laver et al. (2020)	Yes	Can't tell (randomised but method unclear)	Yes (though ITT/imputation unclear?)	P – No<br>I – No<br>A - Yes	Yes – (Small differences re: gender but otherwise similar)	Yes	Yes	Yes	Small benefit but no adverse effects	Trial compared same intervention delivered in two different ways. Majority of outcomes relied on carer report (and may have introduced bias). Cannot discount possible bias as single-blind.
Martín-Martín et al. (2014)	Yes	Yes	Yes	P – No<br>I – No<br>A - Yes	More men in control group but otherwise similar	Control group received 30 minute briefing, otherwise the same	Yes	Yes	No harms found; evidence of benefit	Cannot discount possible bias as single-blind. Note OT was not included within the hospital protocol for hip fracture, so 30-minute briefing given to control group is not TAU.
Sturkenboom et al. (2014)/ (2016)	Yes	Yes	Yes	P – No<br>I – No<br>A - Yes	Yes	No OT for control group at all ?is this usual treatme	Yes	Yes	No harms but minimal improvement to carer outcomes	Intervention appeared to be vs. TAU but control group was reported to have no occupational therapy during the study period – unclear if this is truly TAU (would community therapy normally see these patients if required? Was this prevented during the study period?) Physical therapy was monitored as a confounding factor

Selected articles	Did the study address a clearly focused research question?	Was the assignment of participants to interventions randomised?	Were all participants who entered the study accounted for at its conclusion?	Blinding – were participants (P), investigators (I) and assessors (A) blinded?	Were study groups similar at the start of the RCT? (Carers)	Apart from the intervention, were each group treated equally?	Were the effects of the intervention reported comprehensively?	Was the precision of the estimate of the intervention/treatment effect reported?	Do benefits of the intervention outweigh the harms/ costs? (Carers)	Appraisal summary/comments
						nt				(as well as levodopa equivalent dose). Cannot discount possible bias as single-blind. Combined with process evaluation paper.
Voigt-Randloff et al. (2011a)/(2011b)	Yes	Yes	Yes	P – No<br>I – No<br>A - Yes	Yes – except for financial status	Yes	Yes	Yes (for group mean differences)	No particular benefit or harm found	Cannot discount possible bias as single-blind. Some measures relied on self-report but did have additional analysis from masked research assistants who assessed recordings of task performance. Control group intervention used. Combined with process evaluation paper.
Wenborn et al. (2021)	Yes	Yes	Yes – Intention to Treat (ITT) not used but data analysed by treatment allocated	P – No<br>I – No<br>A - Yes	Yes – mild difference in gender ratios between groups but otherwise very similar	Yes	Yes	Yes	No particular benefit or harm found	Cost-effectiveness of COTID-UK has been investigated elsewhere, as has intervention fidelity and qualitative experiences relating to study participation. Intervention compared to treatment as usual (TAU).
Pilot/feasibility studies										
de Oliveira et al. (2018) **PILOT**	Yes	Yes	Yes	P – No<br>I – No<br>A - Yes	Slightly more males and non-relatives in intervention group	Control group received psychoeducation group sessions	Yes	Yes	No harms reported, some benefits but small sample size (pilot study)	All outcomes reported from pilot studies must be treated with caution. N=11 in intervention group.
Novelli et al. (2018)	Yes	Yes	Yes	P – No<br>I – No	Above average	Yes	Yes	Yes	No harms reported,	All outcomes reported from pilot studies must be treated with caution. N=15 in

Selected articles	Did the study address a clearly focused research question?	Was the assignment of participants to interventions randomised?	Were all participants who entered the study accounted for at its conclusion?	Blinding – were participants (P), investigators (I) and assessors (A) blinded?	Were study groups similar at the start of the RCT? (Carers)	Apart from the intervention, were each group treated equally?	Were the effects of the intervention reported comprehensively?	Was the precision of the estimate of the intervention/treatment effect reported?	Do benefits of the intervention outweigh the harms/ costs? (Carers)	Appraisal summary/comments
**PILOT**				A - Yes	education levels for participating carers ?generalisability				some benefits but small sample size	intervention group.
Jeon et al. (2020) **PILOT**	Yes	Yes	Yes	P – No<br>I – No<br>A - Yes	Yes	Control group offered a book and film vouchers	Yes	Yes	No harms reported, some evidence of benefits	Confidence intervals used contributed to uncertainty in relation to results interpretation. All outcomes reported from pilot studies must be treated with caution. N=9 in intervention group.
O'Connor et al. (2019) **FEASIBILITY/PILOT**	Yes	Yes – exact method of randomisation not clear	Yes	P – No<br>I – No<br>A - Yes	Intervention group slightly older and more males. Otherwise similar	Control group received telephone contacts over course of study	Yes	Yes	No harms reported, some benefits but small sample size	All outcomes reported from pilot studies must be treated with caution. N=9 in intervention group.
Wesson et al. (2013) **FEASIBILITY/PILOT**	Yes	Yes	Yes	P – No<br>I – No<br>A - Yes	Yes	Yes	Largely, some measures missing at follow up	Yes	No harms reported, burden doubled in intervention group	All outcomes reported from pilot studies must be treated with caution. N=11 in intervention group. Intervention vs. TAU. May have benefited from capture of qualitative data. ?burden increase for carers caused by intervention or incidental.

Table C: Summary of quality appraisal using CASP tool for economic evaluations.

Selected articles	Was a well-defined question posed?	Was a comprehensive description of the competing alternatives given?	Does the paper provide evidence that the programme would be effective?	Were the effects of the intervention identified, measured and valued appropriately?	Were all important and relevant resources required and health outcome costs for each alternative identified, measured in appropriate units and valued credibly?	Were costs and consequences adjusted for different times at which they occured?	Was an incremental analysis of the consequences and cost of alternatives performed?	Was an adequate sensitivity analysis performed?	Are the costs translatable to your setting (UK)?	Appraisal summary/comments
Gitlin et al. (2010)	Yes	Evaluated costs of intervention only	Yes	Yes	N/A	N/A	Intervention vs. control	Yes	Unclear	Note: outcomes of analysis are reported in main paper and Table 2.
Rahja et al. (2020)	Yes	Examined if intervention would be of net benefit to society	Yes	Yes	Yes	Yes	Intervention vs. normal practice	Yes	Unclear	Note: outcomes of analysis are reported in main paper and Table 2.

Below: Individual amended Mixed Methods Appraisal Tools (MMAT) completed for studies deemed less appropriate for CASP tools (non-relevant fields from tool deleted for brevity).

1) Allan et al. (2019)

Category of study designs	Methodological quality criteria	Responses			
		Yes	No	Can't tell	Comments
Screening questions (for all types)	S1. Are there clear research questions?	X			
	S2. Do the collected data allow to address the research questions?	X			
	Further appraisal may not be feasible or appropriate when the answer is 'No' or 'Can't tell' to one or both screening questions.				
1. Qualitative	1.1. Is the qualitative approach appropriate to answer the research question?	X			
	1.2. Are the qualitative data collection methods adequate to address the research question?	X			Authors justify use of FG vs interviews
	1.3. Are the findings adequately derived from the data?	X			
	1.4. Is the interpretation of results sufficiently substantiated by data?	X			Very detailed
	1.5. Is there coherence between qualitative data sources, collection, analysis and interpretation?	X			
3. Quantitative non-randomized	3.1. Are the participants representative of the target population?	X			
	3.2. Are measurements appropriate regarding both the outcome and intervention (or exposure)?	X			
	3.3. Are there complete outcome data?	X			For purpose of study
	3.4. Are the confounders accounted for in the design and analysis?	X			
	3.5. During the study period, is the intervention administered (or exposure occurred) as intended?	X			Largely, and well-reported, but some issues using GAS, low evidence of carer support and other issues
5. Mixed methods	5.1. Is there an adequate rationale for using a mixed methods design to address the research question?	X			Very well explained rationale.
	5.2. Are the different components of the study effectively integrated to answer the research question?	X			
	5.3. Are the outputs of the integration of qualitative and quantitative components adequately interpreted?	X			
	5.4. Are divergences and inconsistencies between quantitative and qualitative results adequately addressed?	X			

	5.5. Do the different components of the study adhere to the quality criteria of each tradition of the methods involved?	X			

2) Ariza-Vega et al. (2020)

Category of study designs	Methodological quality criteria	Responses			
		Yes	No	Can't tell	Comments
Screening questions (for all types)	S1. Are there clear research questions?	X			
	S2. Do the collected data allow to address the research questions?	X			
	Further appraisal may not be feasible or appropriate when the answer is 'No' or 'Can't tell' to one or both screening questions.				
3. Quantitative non-randomized	3.1. Are the participants representative of the target population?	X			
	3.2. Are measurements appropriate regarding both the outcome and intervention (or exposure)?	X			
	3.3. Are there complete outcome data?	X			
	3.4. Are the confounders accounted for in the design and analysis?	X			
	3.5. During the study period, is the intervention administered (or exposure occurred) as intended?	X			
5. Mixed methods	5.1. Is there an adequate rationale for using a mixed methods design to address the research question?	X			Use of open-ended questions justified
	5.2. Are the different components of the study effectively integrated to answer the research question?	X			
	5.3. Are the outputs of the integration of qualitative and quantitative components adequately interpreted?	X			
	5.4. Are divergences and inconsistencies between quantitative and qualitative results adequately addressed?	X			
	5.5. Do the different components of the study adhere to the quality criteria of each tradition of the methods involved?			X	

3) Clemson et al. (2020) – note Culph et al. (2020) is a linked mixed methods study (examines same intervention from perspectives of interventionists)

**Category of study designs**	**Methodological quality criteria**	**Responses**			
		Yes	No	Can't tell	Comments
Screening questions<br><br>(for all types)	S1. Are there clear research questions?	X			
	S2. Do the collected data allow to address the research questions?	X			Compared outcomes to original COPE RCT – "hybrid implementation-effectiveness study"
	Further appraisal may not be feasible or appropriate when the answer is 'No' or 'Can't tell' to one or both screening questions.				
3. Quantitative non-randomized	3.1. Are the participants representative of the target population?	X			
	3.2. Are measurements appropriate regarding both the outcome and intervention (or exposure)?	X			
	3.3. Are there complete outcome data?	X			For purpose of study
	3.4. Are the confounders accounted for in the design and analysis?	X			
	3.5. During the study period, is the intervention administered (or exposure occurred) as intended?	X			Detailed information re: fidelity and implementation available

4) Cornelis et al. (2018)

Category of study designs	Methodological quality criteria	Responses			
		Yes	No	Can't tell	Comments
Screening questions (for all types)	S1. Are there clear research questions?	X			
	S2. Do the collected data allow to address the research questions?	X			
	Further appraisal may not be feasible or appropriate when the answer is 'No' or 'Can't tell' to one or both screening questions.				
3. Quantitative non-randomized	3.1. Are the participants representative of the target population?	X			However relatively small sample size (n=30)
	3.2. Are measurements appropriate regarding both the outcome and intervention (or exposure)?	X			May have benefitted from qualitative feedback
	3.3. Are there complete outcome data?	X			
	3.4. Are the confounders accounted for in the design and analysis?			X	
	3.5. During the study period, is the intervention administered (or exposure occurred) as intended?	X			

5) Culph et al. (2020)

Category of study designs	Methodological quality criteria	Responses			
		Yes	No	Can't tell	Comments
Screening questions (for all types)	S1. Are there clear research questions?	X			Very clearly stated
	S2. Do the collected data allow to address the research questions?	X			Lower number of nurses recruited but reflected intervention
	Further appraisal may not be feasible or appropriate when the answer is 'No' or 'Can't tell' to one or both screening questions.				
1. Qualitative	1.1. Is the qualitative approach appropriate to answer the research question?	X			
	1.2. Are the qualitative data collection methods adequate to address the research question?	X			
	1.3. Are the findings adequately derived from the data?	X			
	1.4. Is the interpretation of results sufficiently substantiated by data?	X			
	1.5. Is there coherence between qualitative data sources, collection, analysis and interpretation?	X			
5. Mixed methods	5.1. Is there an adequate rationale for using a mixed methods design to address the research question?	X			Used to enhance researcher understanding of team dynamics. Note no direct oversight by research team when diagrams drawn.
	5.2. Are the different components of the study effectively integrated to answer the research question?	X			
	5.3. Are the outputs of the integration of qualitative and quantitative components adequately interpreted?	X			
	5.4. Are divergences and inconsistencies between quantitative and qualitative results adequately addressed?	X			
	5.5. Do the different components of the study adhere to the quality criteria of each tradition of the methods involved?				N/A. Researchers used novel diagrams to explore

					question

6) Morency et al. (2020)

Category of study designs	Methodological quality criteria	Responses			
		Yes	No	Can't tell	Comments
Screening questions (for all types)	S1. Are there clear research questions?	X			
	S2. Do the collected data allow to address the research questions?	X			Only one OT offered treatment - ?scalability and replicability
	Further appraisal may not be feasible or appropriate when the answer is 'No' or 'Can't tell' to one or both screening questions.				
1. Qualitative	1.1. Is the qualitative approach appropriate to answer the research question?	X			
	1.2. Are the qualitative data collection methods adequate to address the research question?	X			
	1.3. Are the findings adequately derived from the data?	X			
	1.4. Is the interpretation of results sufficiently substantiated by data?	X			Table of quotes provided
	1.5. Is there coherence between qualitative data sources, collection, analysis and interpretation?	X			
4. Quantitative descriptive	4.1. Is the sampling strategy relevant to address the research question?	X			
	4.2. Is the sample representative of the target population?	X			
	4.3. Are the measurements appropriate?	X			
	4.4. Is the risk of nonresponse bias low?			X	Low response rate for x1 surveu (hospital admission)
	4.5. Is the statistical analysis appropriate to answer the research question?				N/A – Feasibility study with limited statistical analysis required
5. Mixed methods	5.1. Is there an adequate rationale for using a mixed methods design to address the research question?	X			
	5.2. Are the different components of the study effectively integrated to answer the research question?	X			
	5.3. Are the outputs of the integration of qualitative and quantitative components adequately interpreted?	X			
	5.4. Are divergences and inconsistencies between quantitative and qualitative results adequately addressed?	X			

	5.5. Do the different components of the study adhere to the quality criteria of each tradition of the methods involved?			X	

7) Nishida et al. (2017)

Category of study designs	Methodological quality criteria	Responses			
		Yes	No	Can't tell	Comments
Screening questions (for all types)	S1. Are there clear research questions?	X			
	S2. Do the collected data allow to address the research questions?	X			
	Further appraisal may not be feasible or appropriate when the answer is 'No' or 'Can't tell' to one or both screening questions.				
3. Quantitative non-randomized	3.1. Are the participants representative of the target population?	X			Pilot – small sample study (n=9 after drop outs)
	3.2. Are measurements appropriate regarding both the outcome and intervention (or exposure)?	X			
	3.3. Are there complete outcome data?	X			
	3.4. Are the confounders accounted for in the design and analysis?			X	E.g. didn't look at impacts of different types of dementia
	3.5. During the study period, is the intervention administered (or exposure occurred) as intended?	X			

8) O'Connor et al. (2020)

Category of study designs	Methodological quality criteria	Responses			
		Yes	No	Can't tell	Comments
Screening questions (for all types)	S1. Are there clear research questions?	X			
	S2. Do the collected data allow to address the research questions?	X			
	Further appraisal may not be feasible or appropriate when the answer is 'No' or 'Can't tell' to one or both screening questions.				
1. Qualitative	1.1. Is the qualitative approach appropriate to answer the research question?	X			
	1.2. Are the qualitative data collection methods adequate to address the research question?	X			
	1.3. Are the findings adequately derived from the data?	X			
	1.4. Is the interpretation of results sufficiently substantiated by data?	X			
	1.5. Is there coherence between qualitative data sources, collection, analysis and interpretation?	X			
3. Quantitative non-randomized	3.1. Are the participants representative of the target population?	X			Note small sample size – pilot study (n=4). Could consider seperating SD and bvFTD cohorts.
	3.2. Are measurements appropriate regarding both the outcome and intervention (or exposure)?	X			
	3.3. Are there complete outcome data?	X			
	3.4. Are the confounders accounted for in the design and analysis?			X	
	3.5. During the study period, is the intervention administered (or exposure occurred) as intended?	X			Pilot study
5. Mixed methods	5.1. Is there an adequate rationale for using a mixed methods design to address the research question?	X			To enhance understandin g of experience of participation
	5.2. Are the different components of the study effectively integrated to answer the research question?	X			
	5.3. Are the outputs of the integration of qualitative and quantitative components adequately interpreted?	X			
	5.4. Are divergences and inconsistencies between quantitative and qualitative results adequately addressed?	X			
	5.5. Do the different components of the study adhere to the quality criteria of each tradition of the methods involved?			X	

9) Pépin et al. (2013)

Category of study designs	Methodological quality criteria	Responses			
		Yes	No	Can’t tell	Comments
Screening questions (for all types)	S1. Are there clear research questions?	X			
	S2. Do the collected data allow to address the research questions?	X			
	Further appraisal may not be feasible or appropriate when the answer is ‘No’ or ‘Can’t tell’ to one or both screening questions.				
3. Quantitative non-randomized	3.1. Are the participants representative of the target population?	X			
	3.2. Are measurements appropriate regarding both the outcome and intervention (or exposure)?	X			
	3.3. Are there complete outcome data?			X	Discussion mentions “qualitative feedback” but not clearly reported in paper?
	3.4. Are the confounders accounted for in the design and analysis?			X	
	3.5. During the study period, is the intervention administered (or exposure occurred) as intended?	X			

10) Pozzi et al. (2019)

**Category of study designs**	**Methodological quality criteria**	**Responses**			
		Yes	No	Can't tell	Comments
Screening questions (for all types)	S1. Are there clear research questions?	X			
	S2. Do the collected data allow to address the research questions?	X			
	Further appraisal may not be feasible or appropriate when the answer is 'No' or 'Can't tell' to one or both screening questions.				
3. Quantitative non-randomized	3.1. Are the participants representative of the target population?	X			Pilot study – small sample size (n=27)
	3.2. Are measurements appropriate regarding both the outcome and intervention (or exposure)?	X			
	3.3. Are there complete outcome data?	X			
	3.4. Are the confounders accounted for in the design and analysis?			X	
	3.5. During the study period, is the intervention administered (or exposure occurred) as intended?	X			
