# Supplementary material for: Occupational therapy interventions for adult informal carers and implications for intervention design, delivery and evaluation: A systematic review
Source: Br J Occup Ther. 2022 Apr 24;86(2):90–100. doi: 10.1177/03080226221079240 (PMC12033786; doi:10.1177/03080226221079240)
Supplement: sj-pdf-3-bjo-10.1177_03080226221079240 – Supplemental Material for Occupational therapy interventions for adult informal carers and implications for intervention design, delivery and evaluation: A systematic review [file sj-pdf-3-bjo-10.1177_03080226221079240.pdf]

Table 2. Summary of eligible papers, including intervention designs and outcomes. Papers are grouped by intervention and then chronological order in terms of publication.

Study	Aim and design	Carer recruitment	Participant characteristics	Intervention (OT element)	Intervention implementation a
Tailored Activity Program (TAP)					
Gitlin et al. (2010a) USA	**Cost-effectiveness study** (linked RCT pre-2010). Aim: To evaluate to cost-effectiveness of the TAP for people living with dementia and their carers	Via media advertising and mailings from social services	N=60 (30 in intervention group, 30 on wait list). **Intervention:** Average age = 62.8 yrs. M=5, F=25. Spouses = 16. **Control:** Average age = 67.9 yrs. M=2, F=28. Spouses = 21. **Patient group:** dementia	**TAP intervention:** OT delivered eight home visits over four months. Included: assessment of patient, carer and home environment; identification of three activities of interest; tailoring these activities to the patient's abilities. Carers were educated about dementia and behaviours and received skill training (e.g. activity simplification, problem-solving and communication skills). Caregivers were also given support to prepare for future deterioration and how to generalise strategies learned **Other Healthcare Professionals (HCPs) involved?** No (OT facilitated)	Average intervention cost = $9 patient-carer interventions). Ca at a cost of $1.10/day and an h $2.37/day (using incremental c effective. **Note:** original RCT (G including improved mastery, se TAP (simplification techniques)
Gitlin et al. (2018) USA	**RCT.** Aim: To determine whether a home-based activity programme (TAP-VA) would reduce behavioural symptoms and functional dependence of veterans with dementia and caregiver burden	Via Veteran Affairs services	N=160 (76 in intervention group). Average age = 72.4 yrs. M=4, F=156. Spouses = 139. **Patient group:** dementia	**TAP-VA intervention:** OT delivered up to eight in-home sessions over four months. Included: assessment of patient, carer and environment (two sessions); production of an assessment report and activity prescriptions, wherein carers learned how to facilitate patient involvement in activities, manage situational distress and understand behavioural symptoms (four sessions); concluded with training carers to simplify activities and use strategies for particular care challenges (two sessions) **Other HCPs involved?** No (OT facilitated)	7.02 sessions (length = 75.5 mi average. 62 dyads completed > effect). **4 months:** Significantly intervention group (-0.07, p = (groups but a trend towards bet significant differences between
de Oliveira et al. (2018) Brazil	**Pilot study.** Aim: To evaluate the efficacy of the tailored activity program–outpatient version (TAP-O) for dementia patients **Control group?** Yes	From community medical centres using media advertising	N=21 carers (12 in intervention group). **Intervention:** M=1, F=11. Average age = 56.8 yrs. **Control**: M=5, F=5. Average age = 60.7 yrs. **Patient group:** dementia	**Adapted TAP intervention:** OT administered in a hospital outpatient setting rather than patient's home. Consisted of eight sessions over three months (condensed programme). Control group participated in psychoeducation group sessions led by an OT **Other HCPs involved?** No - a psychiatrist helped administer outcome measures but did not facilitate the intervention.	Significantly reduced carer bur control group: 20.40 to 21.90 *p*
Novelli et al. (2018) Brazil	**Pilot study.** Aim: To evaluate the effects of the Tailored Activity Program-Brazilian version (TAP-BR) on behavioural symptoms and quality of life in persons with **dementia** and caregiver burden **Control group?** Yes	Media announcements	N=15 carers per group (30 total). M=2, F=28. Average age = 65.97 yrs. **Patient group:** dementia	**TAP intervention** (see above): delivery appeared consistent with original RCT **Other HCPs involved?** No (OT facilitated)	Carer quality of life significantl to 35.73, *p* = 0.02). Caregiver b months (but it was not reduced = 0.5, control: 32.47 to 35.33, *p*
O'Connor et al. (2019) Australia	**Feasibility study.** Aim: To explore the feasibility of implementing the Tailored Activity Program with a cohort of people with frontotemporal	Via a dementia research group, memory clinic and magazine advert	N=20 (9 in intervention group). **Intervention:** M=2, F=7, average age=59. **Control:** M=6, F=5, average age	**TAP intervention** (see above): delivery appeared consistent with original RCT **Other HCPs involved?** No (OT facilitated)	**On average:** 1) 7.44 home visit spent on activity materials. All intervention strategies. 96.3% were still used by sessions 7-8. "doing things" or feeling "on du increasingly useful as sessions **Qualitative themes included:** c *being in control, positive intera*

Study	Aim and design	Carer recruitment	Participant characteristics	Intervention (OT element)	Intervention implementation a
	dementia and their carers (dyads) **Control group?** No		= 66. Spouses = 18, ex-spouses = 1, daughters = 1. **Patient group:** frontotemporal dementia		*importance of this)*; barriers to *engage with process, reluctanc is unsuccessful);* and strategies *used, how these developed)*.
Community Occupational Therapy in Dementia (COTiD)					
Van't Leven et al. (2011) The Nether-lands	**Qualitative interview study.** Aim: To define barriers to and facilitators for implementing the Community Occupational Therapy in Dementia (COTiD) guideline for people with dementia and their carers	OTs recruited from original RCT	N=17 occupational therapists. Average age = 48 yrs (range = 35-60 yrs). **Patient group:** dementia	**COTiD intervention**: OT delivered 10x 60-minute sessions over five weeks. Included: diagnostics and goal setting, identification of activities care and patient wanted to work on, modification of the home environment, identification of potential compensatory strategies to facilitate patient involvement in daily activities (four sessions); teaching the patient how to improve performance of activities and carer training (six sessions). Carer support included: training in cognitive and behavioural interventions, effective supervision and problem solving; practical support; emotional support; and coping strategies **Other HCPs involved?** No (OT facilitated)	**Occupational therapists:** Ident *intervention or dementia itself) instruments, may take longer t* feasibility *(difficult using some narrative interviews for treatm* Facilitators: positivity about CO *intervention phases and carer i to promote in contacts with ph and other departments and po* **physicians:** Barriers: low know OTs; financial resources. Facilit COTiD intervention.
Voigt-Radloff et al. (2011a); (2011b) Germany	**RCT.** Aim: To compare the benefits and harms of a 10-session community occupational therapy programme for patients with Alzheimer's disease with the impact of a one session consultation at home in German routine healthcare	5 memory clinics, 1 municipal hospital and a private neurology practice	N=141 (71 in intervention group). **Intervention:** M = 24, F=47, average age= 64.9. **Control:** M=17, F=53, average age=64.5. Spouses = 80, children = 52, other = 9. **Patient group:** Alzheimer's disease	**COTiD intervention**: OT delivered two 60-minute sessions per week for 10 weeks. Included: assessment of patient, carer and their environment; identification of therapeutic goals (3-4 sessions); treatment phase (5-6 sessions), including environmental adaptation, compensatory strategies and adaptation of activities. Carers received practical and emotional support and training in effective supervision, problem solving, facilitating activities and coping strategies via cognitive-behavioural strategies **Other HCPs involved?** No (OT facilitated)	61 (86%) dyads completed the intervention as: 5 hindering, 15 significant differences on outco suggested several potential exp quality/completion of therapeu patients was better than the or The latter related to: carer not sessions, unwillingness to try so family and carer.
Donkers et al. (2018) The Nether-lands	**RCT.** Aim: To describe the lessons learned from a failed trial which aimed to assess the effectiveness of the Social Fitness Programme (SFP) developed to improve social participation in community-dwelling older people with cognitive problems and their carers	Via general practices, memory clinics, home care and social welfare organisation s and meeting places for people with dementia and their carers	N=17 (8 in intervention group). Note: study did not meet recruitment targets and was ended early. **Patient group:** older people with cognitive impairments (non-specific diagnosis)	**Social Fitness Programme (SFP) intervention:** Modelled on COTiD with additional interdisciplinary components, including: interdisciplinary home visits, discussion between professionals regarding the intervention plan, PT delivery of the Coach2Move programme, and practical support from the welfare professional. Carer-specific elements included: coaching carers in problem solving, compensatory strategies and coping skills, education and advice on dementia, and help to facilitate increased social participation for both patient and carer **Other HCPs involved?** Yes - a physiotherapist (PT) assessed and provided treatment if appropriate (onward referral by OT); a welfare professional (e.g. social worker) if needed for social support	The trial ended early due to rec Recruitment difficulties likely d participation and (b) referring p carers' pre-existing burden - Ad sufficient. - **Barriers to interver** burden; unwillingness of the pa the intervention; interdisciplina up experience with the interve social activities. - **Facilitators to** accept support and contribute collaboration during the interve - All but one interviewed partic not have all personal goals forn Concluded that the SFP did not intervention on social interven delivered as intended; and 3) to patient and carer whilst being c
Pozzi et al. (2019) Italy	**Pilot study.** Aim: To assess the applicability in Italy of the COTID programme on occupational performance of	Convenienc e sample of people on COTiD programme	N=27. Average age = 56.7 yrs, M=9, F=18. **Patient group:** dementia	**COTiD intervention**: OT delivered 10 sessions of home-based OT. First four sessions bi-weekly, including assessment of the patient and goal setting. Sessions 5-10 focused on facilitating improved functional performance, including environmental modification, compensatory strategies, problem-solving and	Only significant difference for c intervention vs. 82.56±12.57 po

Study	Aim and design	Carer recruitment	Participant characteristics	Intervention (OT element)	Intervention implementation a
	people with dementia and their caregivers (COTiD-IT programme) **Control group?** No			education/training for carers **Other HCPs involved?** No (OT facilitated)	
Wenborn et al. (2021)/ Burgess et al. (2020) UK	**Mixed method RCT.** Aims: To estimate the clinical effectiveness of COTiD-UK relative to treatment as usual; To examine the experiences of people with mild to moderate dementia, their family carers and occupational therapists, of taking part in the COTiD-UK intervention	Via NHS services (primarily memory services), voluntary and charitable bodies and a dementia research portal; qualitative study used purposive sampling from RCT	N=468 (249 in intervention group). Average age = 69.1 yrs, range: 29-94 yrs. M=23%, F=77%. Spouses = 72.6%, adult children = 22.2%. N=22 carers completed qualitative study; Age range = 38-88 yrs. Spouses = 18, adult children = 4. **Patient group:** dementia	**COTiD intervention**: OT delivered up to 10 hours of therapy, delivered flexibly, over 10 weeks. OT completed assessment of each member of the dyad and home environment, then facilitated goal setting. Dyads were then supported to achieve goals: for some carers this entailed development of problem-solving and coping strategies. Evaluation regarding goal achievement was completed in the final session **Other HCPs involved?** No (OT facilitated)	**Intervention fidelity:** moderat intervention dyads did not rea average of 4.09 goals were set or partially achieved. Goals not difficulties accessing other serv secondary outcomes similar be UK was more effective than tre more appropriate ways to mea Dyads: 1) appreciated being ab achieve goals: information pro referrals. 2) spoke about facto delays in accessing local servic and communication skills. Inter dyads; others appreciated step
Care of Persons with Dementia in their Environments (COPE)					
Gitlin et al. (2010b) USA	**RCT.** Aim: To test a nonpharmacologic intervention for people living with dementia and their carers that realigns environmental demands with patient capabilities	Media adverts? and mailings via social agencies	N=237 dyads (outcome data collected from 209 at 4 months 102 in intervention group). Average age = 62.2 yrs. M=28, F=186. **Patient group:** dementia	**COPE intervention**: OT delivered up to 10 sessions over four months. Initial sessions: assessment of patient, carer and home environment; identification of caregiving challenges; formulation of action plans ("COPE Prescriptions"). Follow up sessions included caregiver education and caregiver training, such as: problem-solving, communication, how to engage patients in activities, task simplification, environmental modification and stress reduction. Final sessions reviewed progress and how to plan ahead for potential future deterioration **Other HCPs involved?** Yes - one session with an advance practice nurse (information provision; screening of patient for health issues and polypharmacy)	Average of 9.31 face-to-face se minutes) and 3.25 telephone s estimated at $537.05 per dyad difference: 0.22, p = 0.002). Im difference: 0.81, p = 0.002). **9-**between groups. Carers report carers reported self-perceived managing behaviours, life feeli to keep patient at home.
Clemson et al. (2020); Culph et al. (2020); Rahja et al. (2020a); Rahja et al. (2020b) Australia	**Hybrid implementation-effectiveness study.** Aim: To evaluate the effects (in terms of implementation, participant experiences, costs and benefits) of an evidence-based program, Care of People with Dementia in Their Environments (COPE), into health services **Control group?** No; compared outcomes to previous trial (Gitlin et al., 2010b)	Via service providers; eligible dyads identified by OTs. Qualitative study: stratified purposive sampling from larger COPE trial	N=104 dyads (85 completed programme). Age: <65 yrs: 30, 65-74 yrs: 22, 75-84 yrs: 45, 85+ yrs: 7. Spouses = 74, parents = 22, siblings = 3, other = 3. **Patient group:** dementia	**COPE intervention**: delivery appeared consistent with original RCT **Other HCPs involved?** Yes - nurses – see above	31 OTs implemented the progr programme three or more tim (range = 3-10). Average of face prescriptions addressed. **Carer**were rated positively (median post-intervention (33.0 pre-int away from paid employment fo social care system would bene carer 'value' rating: 3.8/4 (4 be appreciated the focus on stres new ways of helping the perso engagement of the patient wit encouraged carers to be less ri for the patient. Carers noted th continue being cared for at ho the community.
Fortinsky et al. (2020)	**RCT.** Aim: To test the COPE intervention for people living with	Via Connecticut Home Care	N=291 (145 in intervention group). Average	**COPE intervention**: delivery appeared consistent with original RCT **Other HCPs involved?** Yes - advanced practice nurse - see above	101 (78%) of dyads completed completing 7 or fewer). **4 mon**the better in the intervention g

Study	Aim and design	Carer recruitment	Participant characteristics	Intervention (OT element)	Intervention implementation a
USA	dementia in a Medicaid and state revenue-funded home and community-based service (HCBS) programme	Program for Elders (CHCPE) – those eligible informed by care managers	age = 61.7 yrs (intervention), 62.7 yrs (control). **Patient group:** dementia		differences between groups. **1** carers. No COPE effects from 4
Laver et al. (2020) Australia	**"Non-inferiority RCT".** Aim: To determine whether delivery of a dyadic intervention for people living with dementia and their carers using telehealth was noninferior to delivery of the same program using traditional face-to-face delivery through home visits	Via memory clinic, aged care wards within a tertiary hospital, community-based dementia education service, local council newsletters	N=63 dyads (31 in adapted intervention/tele health group). Average age = 70.66 yrs (control), 69.47 yrs (adapted intervention). M=15, F=48. **Patient group:** dementia	**Adapted COPE intervention:** Altered schedule to fewer, longer sessions – OT delivered eight sessions over up to 16 weeks, approximately 60 minutes each. Home visit group received all sessions face-to-face. Telehealth group received first two as home visits, the rest via videoconferencing **Other HCPs involved?** No – which is different to usual COPE, (has two sessions with a nurse). Patient instead assessed by the referring service	**Implementation:** Time spent d minutes for home visit group v significantly reduced (255.9 mi **Carers:** No statistically significa measure, the Caregiving Maste confidence interval). No signific measures. Overall no evidence to face-to-face delivery.
Other interventions					
Lam et al. (2010) Hong Kong	**RCT.** Aim: To evaluate a case management (CM) model for people with mild dementia	Via psychogeria tric outpatient and memory clinics at a teaching hospital	N=102 (59 in intervention group). **Intervention**: M=14, F=45. **Control:** M=13, F=30. Ages not provided. **Patient group:** dementia	**Case management**, consisting of OT: assessment of patient and carer; advice on safe performance of self-care, environmental modification, behavioural management, communication techniques; training for carers on home-based cognitive stimulation (reinforced by home visit and phone calls); follow-up at hospital clinics; encouragement for participants to be registered with local support services; accessibility via a hotline and interlinking with other care professionals on behalf of participants **Other HCPs involved?** No (OT facilitated)	Median number of follow-ups **4 months:** No significant chang Questionnaire (GHQ) scores. **1** quality. Increased GHQ score i <0.05) but not in control group case management group – spe months.
Eames et al. (2013) Australia	**RCT.** Aim: To evaluate the effects of an education package which utilised tailored stroke information and reinforcement opportunities on the knowledge, health and psychosocial outcomes of stroke patients and carers	Two acute stroke units	**Intervention** N=31 carers. M=32, F=39, average age = 55.2 years yrs. **Control** N=30 carers. M=31, F=36, average age = 61.4 years yrs. Genders combined (patients and carers). **Patient group:** stroke	**Tailored information provision** by OT for stroke patients/carers with reinforcement via a) telephone contact up to three times post-discharge (monthly intervals) and b) a telephone number participants could call with questions. Telephone support included discussion of useful strategies (e.g. breaking down tasks), correcting misinformation and encouraging healthy behaviours **Other HCPs involved?** MDT helped identify eligible participants but not involved in intervention delivery	On average, the written compo to someone via telephone post between groups for stroke kno
Pépin & King (2013) Australia	**Pilot study.** Aim: To examine measure the effectiveness of the Collaborative Care Skills Training workshops for carers of people living with eating disorders and their transferability to Australian services **Control group?** No	Via eating disorder services, private practitioner s and newspaper adverts	N = 15 (M = 4, F = 11). All mothers/fathers of an adult ED patient (8 were mother/father dyads of the same patient). Average age = 51.29 yrs. **Patient group:** eating disorders	**Collaborative Care Skills Training workshops**. OTs co-facilitated a programme of workshops across six consecutive weeks (2.5 hour sessions). Sessions consisted of education, skill-training (e.g. motivational interviewing, functional analysis, coping strategies, problem solving), and 'homework' tasks **Other HCPs involved?** A clinical psychologist co-facilitated the workshops	Non-significant decrease in: ps maladaptive coping strategies a patient). Significant increase in vs. 44.87 post-programme, *p* = behaviour (mean: 8.79 at basel No change to self-rated guilt o belief the patient could change highly).

Study	Aim and design	Carer recruitment	Participant characteristics	Intervention (OT element)	Intervention implementation a
Wesson et al. (2013) Australia	**Feasibility study.** Aim: To conduct a pilot randomized control trial exploring the design and feasibility of a novel approach to fall prevention for people with mild dementia living in the community **Control group?** Yes	Via a memory disorder/ cognitive disorder/ aged care clinic and a dementia service network	N = 22 (11 in each group). Ages not provided. M = 3, F = 8 (both groups). Relationship to the patient not stated. **Patient group:** dementia	**Tailored 12-week programme to reduce falls.** OT component: six home visits and three telephone contacts. OT completed the Westmead Home Safety Assessment to assess for environmental fall hazards, provided small aids (e.g. sensor lights) and a home safety booklet that provided recommendations tailored to the patient's cognitive ability. OT discussed behavioural issues with carers and provided strategies e.g. task simplification and/or education on patient's abilities **Other HCPs involved?** Physiotherapist prescribed, progressed and monitored adherence to strength and balance exercises for the patient (5 home visits)	Mean number of home visits: 1 (average length = 12.9 minutes home safety recommendations participants reported that they reported. **Carers:** Increased bu (approximately twice that of co routines and briefer instructio
Martín-Martín et al., (2014) Spain	**RCT.** Aim: To examine the effectiveness of an occupational therapy intervention program in reducing emotional distress in informal caregivers of hip fracture patients	Within 24hrs of admission for hip fracture	N=93 carers per group (184 total). **Intervention:** M=11, F=82, average age: 51.6 yrs. **Control:** M=25, F=68, average age = 57.25 yrs. **Patient group:** hip fracture	**Pre-discharge carer educational training programme**: OT delivered training in: manual handling techniques, positioning, facilitating ADLs, and advice on aids and adaptations. Structure: 30-minute educational briefing (control and intervention groups), 45-minute training session and twenty-minute consolidation sessions (intervention only). Written and graphic summaries of the programme were provided **Other HCPs involved?** No (OT facilitated)	**One month:** Carer anxiety incr group (intervention: 26.9% to 2 decreased to a greater extent i 32.1%). Depression gradually d group (one month: interventio intervention 14%, control 25.9 groups.
Sturkenboom et al. (2014); Sturkenboom et al. (2016) The Netherlands	**RCT.** Aim: To assess the efficacy of an occupational therapy intervention in improving daily activities of patients with Parkinson's disease and to evaluate fidelity, treatment enactment and the experiences of participants to identify factors that affect intervention delivery and benefits	From 10 hospitals in nine different regions	N=180 (117 in intervention group). **Intervention:** average age = 67 yrs, M=37, F=80. **Control:** average age=65 yrs, M=21, F=42. **Patient group:** Parkinson's disease	**OTiP trial**: OT delivered a tailored programme (maximum of 16 hours over 10 weeks). Predominantly focused on the patient but carer needs in supporting patient's ADL engagement were assessed and addressed if needed. Consisted of: diagnostic phase (weeks 1-2), goal setting/treatment planning (week 2) and a therapeutic phase (individualised interventions for the patient and carer delivered, weeks 3-10). Carer-specific elements included information provision (e.g. about the disease, aids and adaptations and possible care resources) and skill training to support and supervise the patient when engaging with ADLs. **Other HCPs involved?** No (OT facilitated)	Each OT treated a median of 7 Mean direct intervention time observation of the patient's ac recipients perceived the freque adequate. Only 36% of carers s 41% felt that their ability to cop Main factors identified as affec treatment dose, 2) carer involv successful treatment. **Carers:** N measures except improved qua difference between groups: 0.0 intervention (mean score 7.9 o
Callahan et al. (2017) USA	**RCT.** Aim: To determine whether collaborative care plus 2 years of home-based occupational therapy delays functional decline for people living with Alzheimer's Disease	Via 10 primary care practices and one senior care practice	N=180 (91 in intervention group). Intervention: Average age = 56 yrs, Spouses = 20 Control: Average age = 59.1 yrs, spouses = 28. **Patient group:** Alzheimer's Disease	**ADMIT trial:** OT delivered three cycles of home-based intervention over two years. Cycle 1: 8 90-minute sessions every other week for 16 weeks. Cycle 2: 8 home visits every four weeks. Cycle 3: eight visits over the full year. Issues between visits were addressed via telephone. Carer concerns were identified using the Occupational Profile Checklist. Carer-specific support focused on education and training: using lists and routines, discussion of communication, education about dementia and associated symptoms, facilitating transfers safely, medication management, and teaching carers how to give instructions and modify patient behaviour. **Other HCPs involved?** No (OT facilitated)	Median completed home visits Average session length = 68.5 **Carers:** No significant differen Health Questionnaire (PHQ-9) PHQ-9 score at 12 months app 4.79, p = 0.06).
DiZazzo-Miller et al. (2017)/ DiZazzo-Miller et	**RCT** (though small sample). Aim: To examine the effectiveness of the Family caregiver	Unclear	N=36 (18 in intervention group). Age range = 18-65+ yrs (no average given,	**Family Caregiver Training Programme (FCTP)**: OT delivered a 2-hour training session consisting of three modules (communication, eating and feeding, nutrition; transferring and toileting; dressing, bathing and grooming). Carers given	Significant improvement in kno intervention group (post-test: in control vs. 84.31, p < 0.001). though evidence of increased c post intervention). No significa

Study	Aim and design	Carer recruitment	Participant characteristics	Intervention (OT element)	Intervention implementation a
al. (2020) USA	Training Program (FCTP) for caregivers of people with dementia		mode = 55-64 yrs category in each group). **Intervention:** M=7, F=11. **Control:** M=2, F=16. **Patient group:** dementia	hands-on demonstrations and practice sessions. Case studies used to reinforce learning. **Other HCPs involved?** Unclear	occupational performance or s for the intervention group (13. quality of life domains. Positive given being clear, holding parti both control and intervention g online learning.
Nishida et al. (2017) Japan	**Pilot.** Aim: To develop a home-visit occupation-based programme and determine its effectiveness for people living with dementia and their carers **Control group?** No	Via two community centres	N=11 (2 drop outs). Average age = 62.5 yrs. M=1, F=9. Spouses = 4, children = 3, other = 2. **Patient group:** dementia	**Occupation-based home visit program:** OT delivered eight 1-hour sessions over eight weeks. Programme included: identification of activities meaningful to the client and carer needs (first two sessions); facilitation of selected activities; teaching carers supervision and communication strategies (including verbal or visual cueing, activity simplification and encouragement of the patient). Last session consisted of re-evaluation of patients and carers **Other HCPs involved?** No (OT facilitated)	Dyads received 8.9 home visits statistically significant (baseline increase in carer scores for the for performance (baseline: 2.6 (baseline: 2.5 vs post-intervent
Cornelis et al. (2018) Belgium	**Retrospective pre-test post-test study.** Aim: To determine whether the multicomponent rehabilitation programme of a memory clinic had positive outcomes for people with dementia and carers **Control group?** No	Memory clinic patients	N=30. Ages and genders not provided. Spouses = 24, children = 6. **Patient group:** dementia	**Multicomponent rehabilitation programme based at a memory clinic:** OTs were involved in delivery of a tailored programme (up to 25 sessions including at least two home visits over one year maximum). OT and psychologist completed initial assessment to establish patient and carer goals, plus psycho-education of carers and rehabilitation of the patient. Carer-specific intervention included: teaching cognitive and behavioural strategies to help support patient autonomy, environmental adaptations and assistive technology. At the end of the programme goal achievement was evaluated **Other HCPS involved?** Yes - a geriatrician, neurologist, social worker and psychologist. The social worker completed at least one counselling session and participants received a 6-monthly visit from the geriatrician or neurologist	22 dyads completed the progra **Carers:** No significant differenc approximately 60% of carers sc end of the programme.
Corvol et al. (2018) France	**Qualitative study using semi-structured interviews.** Aim: To explore the usefulness of specialized Alzheimer teams (SATs) as viewed by the patient and their main carer	Purposive sample of people receiving support from a specialized Alzheimer team (SAT)	N=13. Average age = 71.3 yrs (range: 56-93). M=4, F=9. Spouses = 11, daughters = 1, nieces = 1. **Patient group:** Alzheimer's Disease	**Specialized Alzheimer Team (SAT):** OTs co-delivered 12-15 sessions (approx. 1 hour) at least weekly for three months. Sessions 1-3: OT initial assessment and formulation of rehabilitation goals (patient and carer). A nursing assistant then followed a treatment plan devised by the OT, which could include environmental adaptation, carer training/education and compensatory strategies. Final assessment completed by the OT **Other HCPs involved?** Yes - 6 nursing assistants, 4 nurses. Nurses assisted in optimizing planned care and obtaining social support	Both patients and carers had fe progressive nature of the disea Positive feedback was received mood, cognition or behaviour; However, not all carers utilised involved. Some carers used the that the programme allowed ca and understanding of, the patie from dyads prompted changes Authors stated that functional (they may rather prioritise pati carer interactions).
Allan et al. (2019) UK	**Feasibility study.** Aim: To develop and investigate the feasibility and acceptability of the DIFRID (Developing an Intervention for Fall-Related Injuries in Dementia) intervention **Control group?** No	An Emergency Department , via paramedics, primary care and other healthcare professional s	N=11. Spouses = 6, children = 2, other = 3. Ages and genders not stated. **Patient group:** dementia	**DIFRID trial:** OT delivered up to four sessions in the patient's home over a 12-week period. Commenced with holistic assessment of patient, home environment and carer needs. An MDT meeting followed to determine goals and facilitate onward referrals if appropriate. A programme of activities was planned and carried out to fulfil selected goals. Planned carer-specific input included: training (getting up after a fall, positive risk, managing patient resistance to engaging), carer assessment and identification of	Poor implementation of carer s attributed to "a lack of explicit Burden score slightly increased 2, follow-up: 29.7, SD 11.9, ran burden/duplication could have Raised concerns about specific they were largely completed).

Study	Aim and design	Carer recruitment	Participant characteristics	Intervention (OT element)	Intervention implementation a
				support services, upskilling with regards to falls management and prevention **Other HCPs involved?** Yes - physiotherapists delivered up to 4 sessions; therapy assistants/assistant practitioners delivered up to 14	
Clare et al. (2019) UK	**RCT.** Aim: To determine whether or not cognitive rehabilitation is a clinically effective and cost-effective intervention for people with mild to moderate Alzheimer's disease or vascular or mixed dementia, and their carers	Via NHS services (including memory clinics and older age psychiatry services), support groups, a charitable organisation and a dementia research network	N = 474 (238 in intervention group). **Intervention:** Average age = 68.5 yrs. M = 75, F = 163. Spouse/partners = 167, children = 58, other = 13. **Control:** Average age = 69 yrs. M = 67, F = 169. Spouse/partners = 164, children = 60, other = 12. **Patient group:** Alzheimer's disease and dementia	**GREAT trial:** OT delivered 10 sessions over three months followed by four maintenance sessions over six months. Following initial assessment of the patient and goal identification, cognitive rehabilitation was used to maximise patient function. Carers assisted patients in working to achieve goals between sessions. Carers-specific components included: discussion of carer well-being and stress, education, identification of strategies to manage stress, signposting to further support. When carers were not directly present they were kept informed regarding session content. **Other HCPs involved?** Yes - a nurse also helped deliver the intervention sessions; the other nine interventionists were OTs	Retention was 94% at 3 month<br>10 sessions, with 70% completi<br>minutes, average = 75.5 minut<br>effectiveness with regards to C<br>effective in relation to function<br>societal costs when Willingness<br>over 9 months per patient was<br>differences for carer outcomes<br>engagement for intervention s<br>relationship may have influenc<br>provided to carers included ed<br>services (including social servic<br>needing to manage patient-car<br>therapeutic relationship with t<br>2) Increased patience with the<br>Appreciating being able to talk<br>other carers also experienced s<br>useful if they reduced caregivi<br>focused and practical viewpoi<br>confidence and independence<br>appreciated the flexibility and
Ariza-Vega et al. (2020) Spain	**Feasibility study.** Aim: To develop and test feasibility for a post-hip fracture inpatient instructional workshop for caregivers of older adults with hip fracture **Control group?** No	Ward nurses (acute hospitals); posters	N = 210 (103 completed outcome measures). Average age = 52.1 yrs. M = 32, F = 70. Children = 74, spouse or partner = 11, grandchild = 3, niece or nephew = 3, other = 12. Patient group: hip fracture	**Post-hip fracture instructional workshop:** OT delivered 60-90 minute workshops consisting of 1) education on hip fracture and recovery (including hip anatomy, pain management, devices to aid ADLs and the home environment) and 2) practicing hands-on skills to facilitate safe manual handling, completion of ADLs and mobility. Balance and strength exercises were also demonstrated. Content was individualized to the needs of participants **Other HCPs involved?** Yes - orthopaedic surgeon and nurse helped develop workshops; PT helped deliver them	>90% of carers answered all th<br>completion. Median utility and<br>scored the workshop 10. **Quali**<br>design; suggestions for improv<br>caring for patients who are les
Jeon et al., (2020) Australia	**Pilot study.** Aim: To test feasibility and potential effects of the interdisciplinary Home-bAsed Reablement Program (I-HARP) for people living with mild cognitive impairment or mild/moderate dementia and their carers **Control group?** Yes	Multiple sites inc. memory and outreach clinics plus public announcem ents in Sydney area	N = 18 (9 carers per group). Average age = 64 yrs, M = 3, F = 15. **Patient group:** dementia and non-specific cognitive impairment	**I-HARP:** Series of OT home visits of 1.5hrs each (OT delivering 5-6 of these), plus ≤A$1000 worth support for home modifications and assistive technology. Components included: assessment of the patient and environment, creation of a tailored multi-disciplinary plan and two individualised carer support sessions **Other HCPs involved?** Yes - nurse delivered 3-4 hours of programme and neuropsychologist delivered 1 hour of programme. 12 sessions in total (including OT element)	Average cost of intervention de<br>technology, which constituted<br>burden in the intervention grou<br>carers in both groups. **12 mon**<br>control group (effect size 1.40)<br>scores. Further increase to car<br>included: carers feeling unable<br>struggling to understand interv<br>included: positive relationships<br>clinician advice being specialis
Morency et al. (2020) USA	**Feasibility** study. Aim: To explore a six-session wellness intervention for caregivers of cancer patients undergoing hematopoietic stem cell transplantation	Via transplant co-ordinators and social workers	N = 20 at baseline, 14 completed intervention. Average age = 59.3 yrs. M = 8, F = 12. Patient group: cancer	**CARE intervention:** OT-facilitated adaptive coping of carers via six sessions focused on self-selected strategies addressing stressors/buffers to wellbeing. Strategies were based within four wellbeing domains (physical, psychological, social and spiritual). After introducing the intervention rationale (session 1), carers selected goals and created action plans to fulfil these with	14 (74%) of carers completed t<br>average of 8.3 goals; the most<br>carers – 56 goals), stress mana<br>carers – 18 goals). Carers acce<br>sessions). Interviews suggeste<br>that the intervention a) was ta<br>changed perspectives or affirm<br>action instead of worrying, an

Study	Aim and design	Carer recruitment	Participant characteristics	Intervention (OT element)	Intervention implementation a
	(HSCT) entitled Ready to CARE (Connect, Actively Relax, and Exercise) **Control group?** No			support and guidance from the OT (sessions 2-6). Carers also offered support to practice selected activities (e.g. meditation). **Other HCPs involved?** No (OT facilitated)	struggling to find time for the i session timings. 50% of carers (patient admission to hospital) respectively).
O'Connor et al. (2020) Australia	**Feasibility study.** Aim: To examine the functional basis of apathetic and disinhibited behaviours in frontotemporal dementia for four patient-carer dyads and to explore the acceptability of a Positive Behaviour Support (PBS) intervention **Control group?** No	Via a frontotemporal dementia research group	N = 4. Average age = 64.4 yrs. M = 1, F = 3. All lived with the patient and were spouses. **Patient group:** frontotemporal dementia	**PBS intervention:** Initial OT assessment was via functional assessment of behaviours and observations of the patient in the home environment. An individualized Positive Behaviour Support (PBS) plan targeting disinhibited or apathetic behaviours was then developed and completed over a 3-month period over 5-7 home visits. Carers received education and skill training to facilitate management of behaviours **Other HCPs involved?** No (OT facilitated)	Average session length = 80 mi least one behavioural support Carers appeared to develop en Carers appeared to feel PBS wa from it. Three felt no changes v strategies "to manage things" v patient apathy (M = −23%, rang range 14% to −57%) decreased
